# Chemotherapy versus alectinib for the treatment of crizotinib-pretreated ALK-positive patients with non small cell lung cancer: A protocol for systematic review and meta-analysis

**DOI:** 10.1097/MD.0000000000029064

**Published:** 2022-03-18

**Authors:** Ting Gao, Chenxi Li, Xiaopeng He, Tao Zhang

**Affiliations:** ^a^ *Department of Respiratory Medicine, XianYang Central Hospital, Shaanxi, China,* ^b^ *Queen Mary School, Nanchang University, Jiangxi, China,* ^c^ *Department of Chest Surgery, XianYang Central Hospital, Shaanxi, China.*

**Keywords:** alectinib, anaplastic lymphoma kinase-positive non-small cell lung cancer, crizotinib-pretreated, meta-analysis, progression-free survival, protocol

## Abstract

**Background:**

There are no evidence-based data in the literature to demonstrate that alectinib shows a clinically relevant advantage over chemotherapy in anaplastic lymphoma kinase-positive non-small cell lung cancer pretreated with crizotinib. Therefore, we designed this systematic review and meta-analysis protocol to reveal whether the safety and efficacy of alectinib are indeed superior to chemotherapy alone in this special group of patients.

**Methods:**

This protocol will be written following the Preferred Reporting Items for Systematic Reviews and Meta-Analyses Protocols statement guidelines. We will search databases from Web of Science, Embase, PubMed, Wanfang Data, Scopus, Science Direct, Cochrane Library from their inception to June 2022, restricting them to human subjects and clinical trials. Outcomes include progression-free survival, central nervous system progression, and incidence of adverse events. Pooled analyses will be calculated using fixed-effect models, whereas random-effect models will be applied in case of significant heterogeneity across studies. Any disagreements will be discussed and resolved in discussions with the third reviewer.

**Results:**

We hypothesized that alectinib would be superior to chemotherapy in patients with anaplastic lymphoma kinase-positive non-small cell lung cancer pretreated with crizotinib.

**Conclusions:**

The review will add to the existing literature by showing compelling evidence and improved guidance in clinic settings.

**OSF registration number:**

10.17605/OSF.IO/PQF53.

## 1. Introduction

Lung cancer is currently the leading cause of cancer death in many countries, accounting for approximately 20% of all cancer deaths. Non-small cell lung cancer (NSCLC) is the most common form of lung cancer, accounting for approximately 85% of all lung cancer cases.^[1]^ Crizotinib, the first small molecule inhibitor of anaplastic lymphoma kinase (ALK), was approved in the United States in 2011 for the treatment of patients with ALK-positive NSCLC. However, most patients with ALK-positive NSCLC who receive first-line crizotinib will progress within 1 year, usually because of insufficient disease control of the central nervous system.^[2-4]^ Frequent crizotinib resistance and poor central nervous system efficacy are troubling for clinicians.

Alectinib, previously used as second-line therapy for crizotinibresistant patients, is now recommended as first-line therapy for ALK-positive NSCLC. Alectinib is a potent ALK tyrosine kinase inhibitor that effectively penetrates the central nervous system.^[5,6]^ At the same time, activity has been demonstrated against several secondary ALK mutations that may arise following crizotinib treatment; however, the mutational spectrum of ALK may become increasingly complex in patients who have been treated with multiple ALK inhibitors.^[7]^ Concomitant TP53 mutations may also affect the efficacy of alectinib, which has previously been associated with adverse outcomes in patients with advanced ALK-positive NSCLC receiving ALK inhibitors.^[8-10]^

There are no evidence-based data in the literature to demonstrate that alectinib shows a clinically relevant advantage over chemotherapy in ALK-positive NSCLC pretreated with crizotinib. Therefore, we designed this systematic review and meta-analysis protocol to reveal whether the safety and efficacy of alectinib are indeed superior to chemotherapy alone in this special group of patients. We hypothesized that alectinib would be superior to chemotherapy in patients with ALK-positive NSCLC pretreated with crizotinib.

## 2. Materials and methods

This protocol will be written following the Preferred Reporting Items for Systematic Reviews and Meta-Analyses Protocols statement guidelines.

### 2.1. Protocol registration

The prospective registration has been approved by the Open Science Framework registries, and the registration number is 10.17605/OSF.IO/PQF53. No ethical approval is required in our study because all analyses will be based on aggregate data from previously published studies.

### 2.2. Searching strategy

We will search databases from Web of Science, Embase, PubMed, Wanfang Data, Scopus, Science Direct, Cochrane Library from their inception to June 2022, restricting them to human subjects and clinical trials. The literature search keywords are as follows: “crizotinib,” “ceritinib,” “alectinib,” “ALK inhibitor,” and “non-small cell lung cancer.” Bibliographies of relevant articles will be also reviewed to identify additional studies. There are no restrictions on the format and language of the publication.

### 2.3. Eligibility criteria

Two authors will independently screen titles and abstracts to identify potentially eligible articles. They then check the full text to determine final inclusion based on inclusion and exclusion criteria. The authors resolve differences by jointly reviewing relevant studies. If no consensus is reached, a third reviewer will serve as the arbiter. If there are multiple reports for the same trial, we will include data from the most recent reference. Case reports, reviews, editorials, letters, or articles not related to our topic are excluded. All articles meeting the following pre-specified PICOS criteria are considered eligible and included: P: patients with ALK-positive NSCLC pretreated with crizotinib; I: treatment with alectinib alone; C: treatment with chemotherapy alone; O: progression-free survival, central nervous system progression, and incidence of adverse events; S: Randomised controlled trials or cohort studies.

### 2.4. Data extraction

To achieve consistency of extracted items, the data extractor will extract data from eligible research samples. The results of the pilot extraction will be discussed between review authors and extractors. Two independent reviewers will extract data using a predefined extraction template that includes the following items: study characteristics such as first author, year of publication, study design, follow-up period; patient demographic information such as number of patients, mean age, and sex ratio. Outcomes include progression-free survival, central nervous system progression, and incidence of adverse events. If necessary, the original authors will be contacted for missing data. The extracted information will be cross-checked by 2 independent reviewers. Any disagreements will be discussed and resolved in discussions with the third reviewer.

### 2.5. Data synthesis and statistical analysis

Dichotomous data will be analyzed using risk ratio with 95% confidence intervals. Pooled analyses will be calculated using fixed-effect models, whereas random-effect models will be applied in case of significant heterogeneity across studies. When no events are observed, 0.5 will be added to both arms of the trial. Statistical heterogeneity will be measured using the *I*^2^ statistic. Meta-regression analyses will be conducted to estimate the extent to which other covariates may have influenced the treatment effects. Sensitivity analyses will be performed to determine the stability of the overall treatment effects. Additionally, publication bias will be assessed using the Begg adjusted rank correlation test and Egger regression asymmetry test. All *P* values will be 2-tailed, and the statistical significance will be set at 0.05. Review Manager software (v 5.4; Cochrane Collaboration) will be used for the meta-analysis.

### 2.6. Risk of bias

The Cochrane risk of bias tool will be independently used to evaluate the risk of bias of included randomized cohort studies by 2 reviewers. The quality will be assessed by using following 7 items: random sequence generation, allocation concealment, blinding of participants and personnel, blinding of outcome assessment, incomplete outcome data, selective reporting, and other bias. A modified version of the Downs and Black tool is adopted to evaluate the quality of nonrandomized cohort studies. The modified version consists of 27 items with a total possible score of 29. A score of ≥ 75% indicates high quality, 60% to 74% indicates moderate quality, and ≤ 60% low quality. Two investigators independently evaluate included studies on the 27 criteria, with any discrepancies resolved by a third independent reviewer.

## 3. Discussion

Since the initial development of ALK inhibitors, subsequent clinical trials on the efficacy of the ALK inhibitors have been published. However, there are no evidence-based data in the literature to demonstrate that alectinib shows a clinically relevant advantage over chemotherapy in ALK-positive NSCLC pretreated with crizotinib. Therefore, we designed this systematic review and meta-analysis protocol to reveal whether the safety and efficacy of alectinib are indeed superior to chemotherapy alone in this special group of patients. We hypothesized that alectinib would be superior to chemotherapy in patients with ALK-positive NSCLC pretreated with crizotinib.

## Author contributions

**Conceptualization:** Chenxi Li, Xiaopeng He.

**Data curation:** Ting Gao, Chenxi Li.

**Formal analysis:** Ting Gao, Chenxi Li.

**Funding acquisition:** Tao Zhang.

**Investigation:** Ting Gao, Chenxi Li.

**Methodology:** Xiaopeng He.

**Resources:** Tao Zhang.

**Software:** Chenxi Li, Xiaopeng He.

**Supervision:** Tao Zhang.

**Validation:** Xiaopeng He.

**Visualization:** Xiaopeng He.

**Writing** - **original draft:** Ting Gao.

**Writing** - **review & editing:** Tao Zhang.
